# Characterization of the complete mitochondrial genome of *Squalus brevirostris* (Squaliformes, Squalidae)

**DOI:** 10.1080/23802359.2019.1660595

**Published:** 2019-09-06

**Authors:** Nan Zhang, Hua-Yang Guo, Liang Guo, Ke-Cheng Zhu, Bao-Suo Liu, Jing-Wen Yang, Dian-Chang Zhang

**Affiliations:** a Division of Aquaculture and Genetic Breeding, South China Sea Fisheries Research Institute, Chinese Academy of Fishery Sciences, Guangzhou, China;; b Key Laboratory of South China Sea Fishery Resources Exploitation and Utilization, Ministry of Agriculture, Guangzhou, China;; c Guangdong Provincial Engineer Technology Research Center of Marine Biological Seed Industry, Guangzhou, Guangdong Province, China

**Keywords:** *Squalus brevirostris*, mitochondrial DNA, conservation genetics, phylogenetic relationship

## Abstract

The complete mitochondrial genome sequence of *Squalus brevirostri* was determined using next-generation sequencing. The whole circular genome is 16,734 bp in length. It contains 37 genes including13 protein-coding genes (PCGs), two ribosomal RNA (rRNA) genes, 22 transfer RNA genes (tRNA), and a control region (D-Loop). The overall nucleotide composition is A: 30.38%, T: 30.73%, G: 24.61%, and C: 14.27%, with an A + T content of 61.11%. The phylogenetic analysis suggested that *S. brevirostris* was closely related to *Squalus montalbani*.

The Shortnose spurdog (*Squalus brevirostris*) is a member of the family Squalidae. It was found in the subtropical and tropical seas, and the distribution extending from waters of southern Japan and South China Sea. Due to anthropogenic over-exploitation and destroy of its natural habitat, wild resources of *S. brevirostris* have dramatically declined. Developing reasonable conservation and utilization measures for *S. brevirostris* requires a deeper understanding of molecular genetics. In addition, lack of phylogenetic studies led limited understanding of evolutionary relationships of species within the family. The complete mitochondrial genomes could provide more informations than single genes, and show genome-level characteristics which are valuable for better understanding of genome evolution and phylogeny (Williams et al. 2014). In this study, we sequenced the complete mitochondrial genome to provide useful molecular markers for population genetics, conservation biology and evolutionary studies of *S. brevirostris.*

A sample of *S. brevirostris.* was collected from South China sea (N19°03′, E115°36′) in September, 2015. This sample (Ssfri-F0054) is currently stored at the Fisheries Museum Specimens of South China Sea Fisheries Research Institute in 95% ethanol. Total genomic DNA from muscle tissue was extracted with the modified CTAB method (Porebski et al. 1997). We assembled the complete genome with Velvet v 1.0 software (Zerbino and Birney 2008), with that of *Squalus formosus* (GenBank: KU951280.1) as initial reference. The gene map of mitochondrial genome of *S. brevirostris* was drawn by OGDRAW1.2 (Lohse et al. 2013) and modified manually.

In this study, the complete mitochondrial genome of *S. brevirostris* is 16,734 bp in size (Gen-Bank: KY111436), which consists of 13 protein-coding genes (PCGs), 2 ribosomal RNA(rRNA) genes, 22 transfer RNA (tRNA) genes, and a control region (D-Loop). The nucleotide composition of *S. brevirostris* mitochondrial genome is A: 30.38%, T: 30.73%, G: 24.61%, and C: 14.27%, which demonstrated an A + T (61.11%) rich feature. Twenty-nine mitochondrial genes are encoded on the H-strand and the remainders are mapped to the L-strand. A total of 14 gaps (67 bp) and three overlaps (21 bp) were identified among the genes. The control region was located between *tRNA^Phe^* gene and *tRNA^Pro^* gene. The 13 identified PCGs have a length varying from 168 to 1833 bp.

A phylogenetic analysis was conducted on eight mitochondrial genomes from Squaliformes. The neighbor-joining phylogenetic tree were constructed by MEGA6 (Tamura et al. 2013).The phylogenetic analysis showed that all representatives of Squalidae were clustered into one monophyletic clade, and *S. brevirostris* is closely related to *Squalus montalbani* (Figure 1). The complete mitochondrial genome of *S. brevirostris* provides essential information for understanding phylogenetic relationships of Squaliformes mitochondrial genome and will be useful for the conservation genetics of this species.

**Figure 1.**
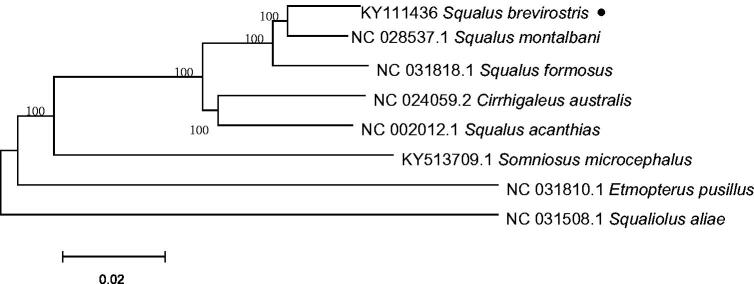
The phylogenetic tree based on eight mitochondrial genome sequences. GenBank accession numbers: *Squalus brevirostris* KY111436, *Squalus montalbani* NC_028537.1, *Squalus formosus* KU951280.1, *Cirrhigaleus australis* NC_024059.2, *Squalus acanthias* NC_002012.1, *Somniosus microcephalus* KY513709.1, *Etmopterus pusillus* KU892588.1, *Squaliolus aliae* NC_031508.1.
